# Supplementary figures and images for: Secretome Analysis of Clavibacter nebraskensis Strains Treated with Natural Xylem Sap In Vitro Predicts Involvement of Glycosyl Hydrolases and Proteases in Bacterial Aggressiveness
Source: Proteomes. 2021 Jan 9;9(1):1. doi: 10.3390/proteomes9010001 (PMC7839047; doi:10.3390/proteomes9010001)

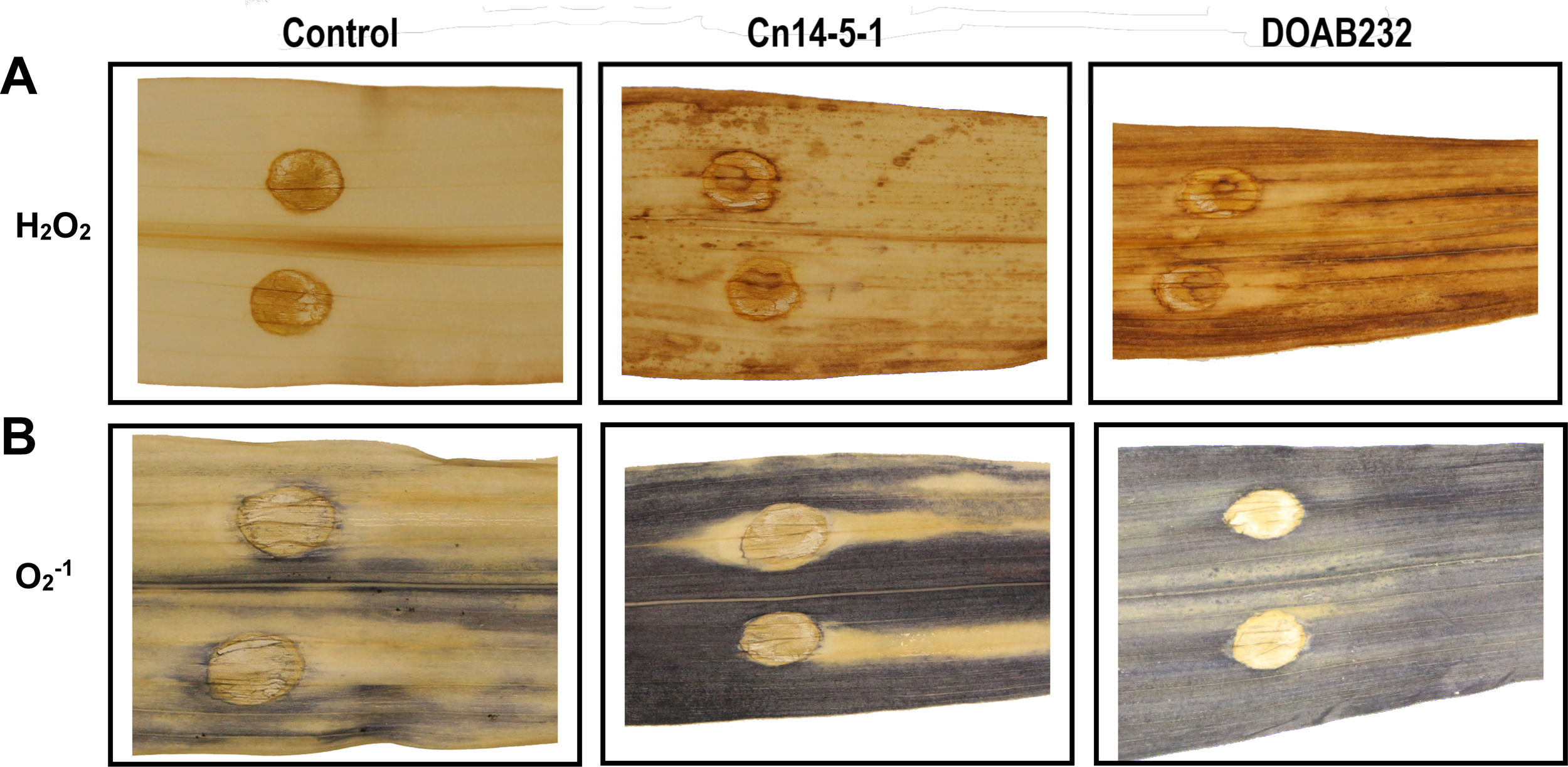

Supplement: Supplementary file 1 [file proteomes-09-00001-s001.zip › SS2-Figure S2.tif]

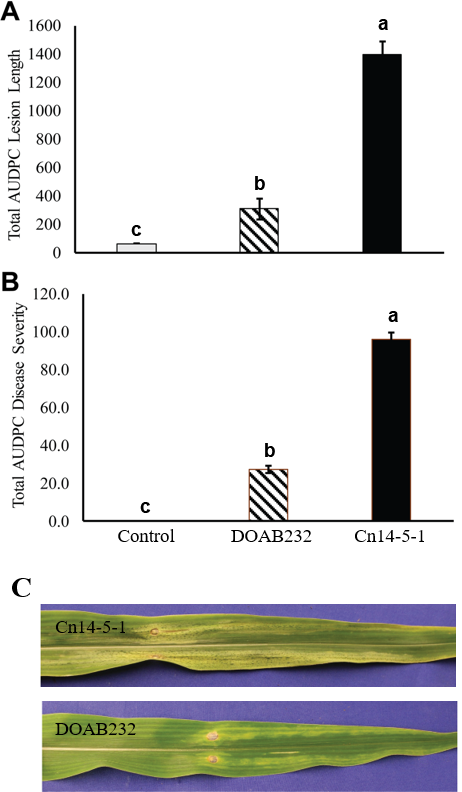

Supplement: Supplementary file 1 [file proteomes-09-00001-s001.zip › Figure S1.tif]
